# Biocompatible Silica-Polyethylene Glycol-Based Composites for Immobilization of Microbial Cells by Sol-Gel Synthesis

**DOI:** 10.3390/polym15020458

**Published:** 2023-01-15

**Authors:** Daria G. Lavrova, Anton N. Zvonarev, Valery A. Alferov, Tat’yana G. Khonina, Elena V. Shadrina, Sergey V. Alferov, Olga N. Ponamoreva

**Affiliations:** 1 Biotechnology Department, Tula State University 1, 300012 Tula, Russia; 2 Federal Research Center “Pushchino Scientific Centre of Biological Research”, G.K. Skryabin Institute of Biochemistry and Physiology of Microorganisms, Russian Academy of Sciences, 142290 Pushchino, Russia; 3 Chemistry Department, Tula State University 1, 300012 Tula, Russia; 4 Laboratory of Organic Materials, I.Ya. Postovsky Institute of Organic Synthesis, 620990 Yekaterinburg, Russia; 5 Laboratory of Ecological and Medical Biotechnology, Tula State University 1, 300012 Tula, Russia

**Keywords:** ORMOSIL composites, PEG, silica, sol-gel, immobilized cells, biocatalysts, biofilter

## Abstract

Biocatalysts based on the methylotrophic yeast *Ogataea polymorpha* VKM Y-2559 immobilized in polymer-based nanocomposites for the treatment of methanol-containing wastewater were developed. The organosilica composites with different matrix-to-filler ratios derived from TEOS/MTES in the presence of PEG (SP_EG_-composite) and from silicon-polyethylene glycol (STP_EG_-composite) differ in the structure of the silicate phase and its distribution in the composite matrix. Methods of fluorescent and scanning microscopy first confirmed the formation of an organosilica shell around living yeast cells during sol-gel bio-STP_EG_-composite synthesis. Biosensors based on the yeast cells immobilized in STP_EG_- and SP_EG_-composites are characterized by effective operation: the coefficient of sensitivity is 0.85 ± 0.07 mgO_2_ × min^−1^ × mmol^−1^ and 0.87 ± 0.05 mgO_2_ × min^−1^ × mmol^−1^, and the long-term stability is 10 and 15 days, respectively. The encapsulated microbial cells are protected from UV radiation and the toxic action of heavy metal ions. Biofilters based on the developed biocatalysts are characterized by high effectiveness in the utilization of methanol-rich wastewater—their oxidative power reached 900 gO_2_/(m^3^ × cycle), and their purification degree was up to 60%.

## 1. Introduction

Over the last decade, organosilica composites (organosilica hybrid materials, ORMOSILs) have found wide application in various areas of human activity due to their better mechanical properties, thermal stability, and endurance compared to polymer materials due to the inclusion of silica filler into the polymer matrix [1,2,3,4,5,6,7]. ORMOSIL is an organically modified sol-gel silica synthesized by the incorporation of organic functional groups/compounds into silane alkoxides or by the addition of organic components to silane alkoxides during sol-gel synthesis [8]. Methods of sol-gel chemistry do not require energy-consuming and expensive equipment; they are environmentally friendly and economically feasible, which is an advantage for the development of technologies of organosilica nanocomposite synthesis [9]. The sol-gel method is used in the engineered and controlled formation of ceramic nanopowders and to fabricate oxide, non-oxide, and composite nanopowders [10,11,12,13]. Two major classes of ORMOSIL depending on the kind of molecular bonds/interactions have been described: class I hybrids and class II hybrids [14,15]. Class I ORMOSILs are hybrid composites that do not contain covalent bonds between organic and inorganic phases, and they could be obtained via the inclusion of organic components, such as various polymers, into the system, leading to the formation of spatial network interactions between the inorganic and organic components of hybrid materials [14,16,17,18]. Mutual permeation of the 3D networks is achieved by simultaneous gelation of organic and inorganic components.

High-molecular organic polymers play the role of a structure-forming matrix for ORMOSIL; one of such polymers is polyethylene glycol (PEG) [19,20,21,22,23,24,25,26,27,28,29,30,31,32,33,34]. PEG has hydrophobic hydrocarbon fragments and hydrophilic oxygen bridges, so this polymer can change its polarity depending on the nature of the solvent or other components in the system [35,36,37,38]. PEG molecules are able to flocculate silica sol particles via the formation of hydrogen bonds between ether atoms of oxygen in PEG and Si-OH groups on the silica surface. It should be noted that, depending on molecular mass and fraction of PEG, different spatial structures of hydrogel are formed, which determine the morphology and final properties of organosilica nanocomposites [27]. Interaction between PEG and silica particles leads to the emergence of novel materials with specific characteristics that differ from the characteristics of their components: hydrogel strength increases due to the volume distribution of silica particles, whereas the elasticity of silica materials rises due to the effect of structure-forming PEG chains. This approach allows for the preparation of elastic hydrogel, flexible rubber, or solid mesoporous glass [15,39,40].

These materials display good stability and biocompatibility [39], and they have a significant potential for various biomedical and biotechnological applications [6,7], which makes them promising matrices for the immobilization of microorganisms [41,42,43,44,45,46,47]. Immobilization of microorganisms in organosilica nanocomposites leads to stabilization of their catalytic activity, providing the opportunity for repeated or continuous use of biocatalysts [43,48,49,50,51,52,53,54]. The biohybrid materials mimic natural unicellular organisms, diatoms, which are capable of forming a protective silica exoskeleton on their surface [55]. Information on the immobilization of whole cells of microorganisms by sol–gel synthesis as well as their use in environmental bioremediation is summarized in the recent reviews [46,56,57,58,59,60].

Usually, only fully hydrolysable alkoxysilane precursors, such as tetraethoxysilane (TEOS), are used as source compounds, which results in a yield of silica with a rigid structure [24,41,61,62,63]. The addition of an alkylalkoxysilane precursor containing a non-hydrolysable Si-C bond makes the yield of flexible organosilica materials (ORMOSIL type 2) possible [62,63,64]. We have earlier studied the possibilities of encapsulation of microorganisms into organosilica composites made of TEOS and methyltriethoxysilane (MTES), as hydrophobic additives, in the presence of PEG via one-step sol-gel synthesis. Methylotrophic yeasts possessing an effective short-chain alcohol oxidation system were used as a biological part of the composite biocatalysts. In these conditions, upon a certain ratio of silane precursors, yeast cells become the centers of formation of the so-called “cell in the shell” architectures [65,66,67,68]. Such shells protect the cells from environmental stress factors (heavy metal ions, UV radiation, and extreme pH values) [65,67], which is important for the development of industrial wastewater purification technologies.

Silicon polyethylene glycolates are used as precursors in the synthesis of organosilica composites for medical and biotechnological applications [69,70,71,72]. Their advantage over the conventional alkoxyl precursors based on monoatomic alcohols is that the hydrophilic polymers do not cause denaturation and/or precipitation of biological macromolecules. A silicon ethylene glycol precursor was used in the process of biomimetic mineralization of polysaccharides, proteins, and synthetic biopolymers [73,74]. However, we could not find information on the use of these precursors for the immobilization of living cells.

Comparative analysis of the effectiveness of biocatalysts based on methylotrophic yeast *Ogataea polymorpha* VKM Y-2559 immobilized into organosilica materials of various compositions was studied in the present work. Immobilization of the microorganisms was carried out by one-stage sol-gel synthesis under basic catalytic conditions, and the following source reagents were used: the first variant (SP_EG_) involved TEOS:MTES in a 15:85 volume:volume ratio and PEG3000 as a structure-forming agent, and the second variant (STP_EG_) was composed of silicon tetrapolyethylene glycol synthesized from TEOS and PEG400 [70].

The goal of the research was to elaborate on biocatalysts based on the methylotrophic yeast *Ogataea polymorpha* VKM Y-2559 immobilized in PEG and silica nanocomposites for the treatment of methanol-containing wastewater.

## 2. Materials and Methods

### 2.1. Materials

Tetraethoxysilane (≥99.9%) and methyltriethoxysilane (MTES, ≥ 99.9%) were purchased from Sigma-Aldrich (St. Louis, MO, USA). Silicon polyethylene glycol (STP_EG_) was synthesized according to the method described earlier [70]. Polyethylene glycol 3000 (PEG3000) from Ferak (Berlin, Germany). All other reagents were of analytical grade, and they were used as received without further purification.

### 2.2. Microorganism Cultivation

The yeast strain *Ogataea polymorpha* VKM Y-2559 was received from the National Collection of the Institute of Biochemistry and Physiology of Microorganisms (Pushchino, Russia). The cultivation of the yeast *Ogataea polymorpha* VKM Y-2559 was carried out according to the standard method described earlier [75]. The yeast biomass was stored in polypropylene test tubes at +4 °C.

### 2.3. Sol-Gel Synthesis of Organosilica Nanocomposites and Encapsulation of Yeast Cells

#### 2.3.1. STP_EG_-Composites

**Figure 1:**
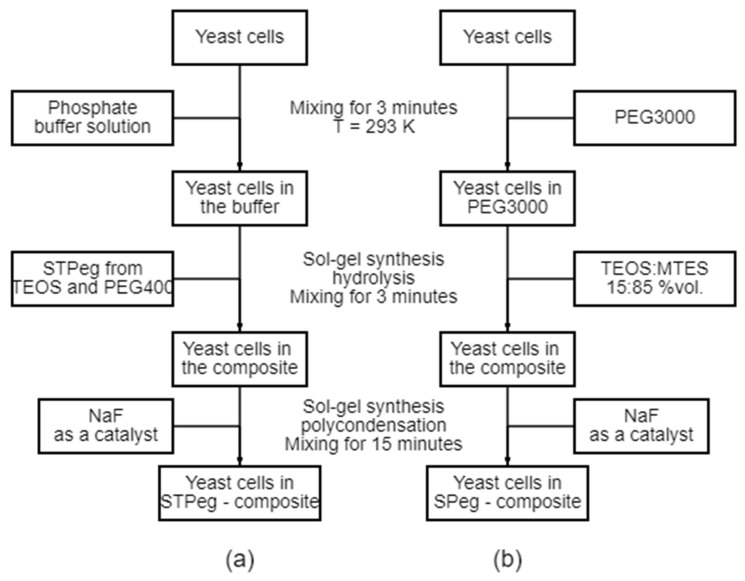
Immobilization of yeast cells in silica-polyethylene glycol-based composites by sol-gel synthesis: (**a**) immobilization of yeast cells in STP_EG_-composite; (**b**) immobilization of yeast cells in SP_EG_-composite.

A quantity of 0.35 cm^3^ of phosphate buffer solution (33 mM, pH 7.6) or 0.35 cm^3^ of yeast suspension (1.3 × 10^9^ CFU/cm^3^, 40 mg cell biomass) in phosphate buffer solution (33 mM, pH 7.6) was added to 0.5 cm^3^ STP_EG_ and mixed for 3 min. Then, 0.025 cm^3^ of the catalytic solution of NaF (0.2 M) was added and stirred for 15 min (Figure 1a). As a result, a biohybrid material was obtained in the form of hydrogels. The absence of specific colonies in the buffer put on an agar plate signified the completion of cell immobilization.

#### 2.3.2. SP_EG_-Composites

A quantity of 0.25 cm^3^ of phosphate buffer solution (33 mM, pH 7.6) or 0.25 cm^3^ of yeast suspension (1.3 × 10^9^ CFU/cm^3^, 40 mg cell biomass) in phosphate buffer solution (33 mM, pH 7.6) was added to 0.1 cm^3^ of 20% PEG3000 solution and mixed for 3 min. Then, 0.5 cm^3^ of the TEOS and MTES mixture (TEOS, 0.075 cm^3^, and MTES, 0.425 cm^3^) was added and mixed again for 3 min. Then, 0.025 cm^3^ of the catalyst solution of 0.02 M NaF was added and stirred for 15 min (Figure 1b). As a result, a biohybrid material was obtained in the form of hydrogels. The absence of specific colonies in the buffer put on an agar plate signified the completion of cell immobilization.

### 2.4. Biocatalyst Bed Preparation for Biofilter Column

Glass beads (3.3 ± 0.3 mm in diameter) served as biofilter bed carriers. The glass beads were immersed in 0.1 M HCl for 2 h before use. Organosilica composites with entrapped yeast (4.375 cm^3^ aliquots) were applied to 150 activated glass beads. The modified beads were transferred into a chromatographic column 2.0 cm in diameter, where bed carriers were 5.0 cm long (short column), or a chromatographic column 1.0 cm in diameter, where bed carriers were 10.0 cm long (long column), cooled for 24 h and washed with buffer (pH = 7.6) until methanol was completely removed. The maximal methanol concentration in wastewater acceptable for biochemical treatment is 220 mg/L. The biofilter was tested for the capacity to treat a model of methanol polluted wastewater (220 mg/L of methanol) in two modes (with and without active aeration) at a flow rate of 0.5 mL/min (0.96 L/h × L_filter bed_). Aeration was performed through a plastic tube 0.5 cm in diameter with 100 perforations (0.4 mm each) placed in the middle of the column with a filter bed inside using an SB-348 air compressor (Sobo, Zhongshan, China) at an air flow rate of 4 L/min. Methanol content in the eluate was monitored by gas chromatography.

### 2.5. Instrumental Analysis

The respiratory activity of immobilized cells in the presence of substrate (methanol) was used as an indicator of their biocatalytic activity. The biohybrid materials (Section 2.3) were placed on the surface of a Clark oxygen electrode. A 0.02 cm^3^ aliquot of hydrogel was applied to a porous fiberglass filter (Whatman GF/A, Sigma-Aldrich, St. Louis, MO, USA) and dried for 15 min at 20 °C. A 3 × 3 mm fiberglass filter fragment with immobilized cells on it was placed on the oxygen electrode surface and fixed with nylon mesh. The entrapped yeast cell respiratory activity was investigated using an EXPERT-001-4.0.1 pH-meter/ion meter/BOD thermo-oximeter (Econix-Expert Ltd., Moscow, Russia) coupled to a personal computer operated by specialized software EXP2PR (Econix-Expert Ltd., Moscow, Russia). The measured parameter (biosensor response) was the maximal rate of oxygen concentration change at the addition of substrates (mg/dm^3^ × min). Dynamic viscosity was measured on a Haake Viskotester 550 (Thermo Fisher Scientific, Waltham, MA, USA) viscometer with a measurement error of ±6%. IR spectrometry was used to calculate the number of Si-O-Si bonds. The IR spectra of solutions of the precursors and sol-gel systems were recorded with the FMS 1201 Fourier IR spectrometer (OOO Monitoring, Saint-Petersburg, Russia) using a horizontal-type multiple attenuated total internal reflection (MATIR) unit with a cadmium selenide prism (resolution 4 cm^–1^). The IR spectra of the samples were recorded 15 min after the start of sol-gel synthesis. The surface morphology of the sample was examined by scanning electron microscopy (SEM). Samples of the yeast cells *Ogataea polymorpha* encapsulated in organosilica composites were fixed at 4 °C for 12 h in 0.05 M sodium cacodylate buffer (pH 6.8) containing 1.5% glutaraldehyde and then post-fixed at 20 °C for 3 h in the same buffer supplied with 1% OsO_4_. After dehydration, the samples were coated with gold (Fine Coat Ion Sputter JFC-1100, Tokyo, Japan) and examined under a scanning microscope JSM-6510LV (JEOL, Tokyo, Japan). To compare the content and localization of cells, hydrogel slices and cells were stained with the fluorescent fungal surface-labeling reagent Calcofluor White M2R (Thermo Fisher Scientific, Waltham, MA, USA). To obtain fluorescent micrographs, thin slices were made using a razor blade. The filter set 49 (Zeiss, Jena, Germany) was used with the excitation maximum at 365 nm and with the emission bandpass at 445 nm. Live and dead cells were revealed using a Live/Dead Yeast Viability Kit (Molecular Probes, Eugene, Oregon, USA). The cells were examined by phase-contrast and fluorescent microscopy in an AXIO Imager A1 (Zeiss, Jena, Germany) with a filter set of 56HE (Zeiss, Jena, Germany) at a wavelength of 450–500 nm for excitation and 512 + 630 nm for emission. An Axiocam 506 camera (Zeiss, Jena, Germany) was used to acquire images. The methanol alcohol content was measured by gas chromatography on a chromatograph, «Crystal 5000.2» (Chromatec, Yoshkar-Ola, Russia), with a flame ionization detector and a capillary column, DB-FFAP (50 m × 0.32 mm × 0.50 µm) (Agilent, Santa Clara, CA, USA). Analysis conditions were as follows: the column oven temperature was 70 °C, the evaporator temperature was 200 °C, the detector temperature was 2–50 °C, and the carrier flow rate of helium was 0.10 dm^3^/h.

## 3. Results and Discussion

### 3.1. Synthesis and Properties of Organosilica Composite Materials

Two reagent systems including PEG differing in molecular mass (PEG3000 in SP_EG_-composite and PEG400 in STP_EG_-composite) were used as a polymeric matrix in the study for nanocomposite synthesis and cell immobilization. This was caused by different methodological approaches in the synthesis of silicon polyethylene glycol [66,68,70]. In addition, filler particles were synthesized from a TEOS:MTES mixture (15:85 v:v ratio) when the SPEG composite was produced. This ratio was selected based on our previous studies on the synthesis of biocompatible hydrogels [65].

**Figure 2:**
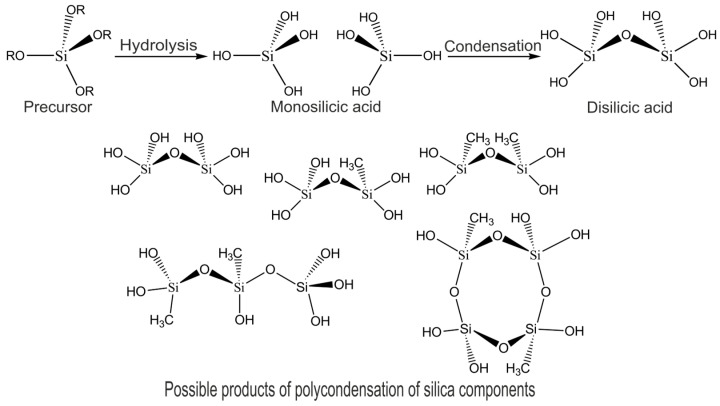
Scheme of sol-gel synthesis of silica compounds and possible products of polycondensation.

Hydrogel composites made of STP_EG_ and SP_EG_ for microbial cell immobilization were obtained under sol-gel synthesis conditions in Na-K-phosphate buffer solution (pH = 7.6) in the presence of a catalytic amount of sodium fluoride. During hydrolysis and condensation of silicic acid monomers in an aqueous environment, oligo- and polysilicic acids are formed as water-insoluble sol particles, which then turn into gel (Figure 2).

The addition of PEG into the reaction mixture led to the formation of organosilica composites, and their structure depended on the ratio of PEG and silica components. Table 1 shows mass ratios of PEG and silica used in the sol-gel synthesis of organosilica composites.

**Figure 3:**
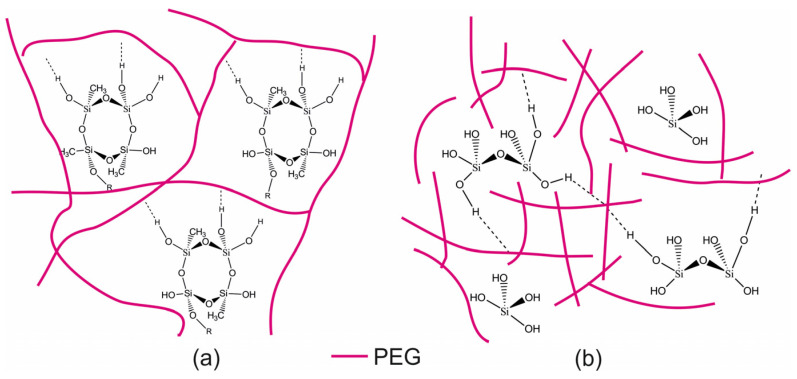
Assumed schematic structure of nanocomposites: (**a**) SP_EG_-composite; (**b**) STP_EG_-composite.

The content of silica in SP_EG_-composite is approximately 32 times higher than that in STP_EG_-composite. This leads to the formation of silica clusters in the organosilica hydrogels of SP_EG_, including ring-shaped structures of [SiO_4_] tetrahedrons, which are similar to the crystals of quartz (Figure 3a). According to the authors of [74], it is typical to distinguish colloidal and polymeric gels for silicon polyethylene glycol precursors. The relatively low content of silica in STP_EG_-composite facilitates the formation of silanol groups (-Si-OH) as centers of attachment of low-molecular-weight PEG400 chains (Figure 3b), but it is insufficient for silica cluster formation. The polymer gels form under these conditions. In addition, a smaller amount of water molecules is localized in organosilica hybrids with higher PEG content due to the formation of a higher number of hydrogen bonds between inorganic silica and organic PEG additives instead of bonds including composite parts and water molecules [76]. The results of studies on the adsorption of methylene blue on olive stone waste supplied with PEG-silica provide indirect confirmation for this proposal. According to them, the dye is adsorbed on this composite to a greater extent, which might be related to the decrease of water adsorption [77]. Another study [78] demonstrated that organosilica hybrids with a high mass fraction of PEG (50% and more) retained less water. These differences in the structure of organosilica source materials are reflected in the morphology of the final composites.

The chemical composition of the solid phases of silicon polyethylene glycol hydrogels without the microbial cells has been determined earlier [74]. The solid phase was separated by exhaustive cold extraction in absolute ethanol. The solid samples obtained after extraction were analyzed by combined thermal analysis, simultaneous quadruple mass spectrometry (QMS), and XRD analysis. The weight loss in the thermal decomposition of the solid phase was 80%, and according to QMS plots, the evolving gases contained large concentrations of CO_2_. It means that up to 80% of the solid phase of the hydrogel corresponds to the organic moieties, which are likely PEG residues. An XRD plot revealed no crystallinity in the solid phase of the gel.

Hydrogel STP_EG_-composites comprise a viscous semitransparent substance (dynamic viscosity 45–80 Pa in the range 2 ≤ y ≤ 20) as well as SP_EG_-composites, which are heterogeneous materials resembling quartz particles stuck to each other with polymer glue. Interactions between PEG and silica/organosilica particles lead to the formation of novel materials with specific properties different from those of separate components: the mechanical strength of the hydrogels rises due to the volume distribution of silica particles, while the elasticity of the silica materials grows due to the structure-forming PEG chains.

**Figure 4:**
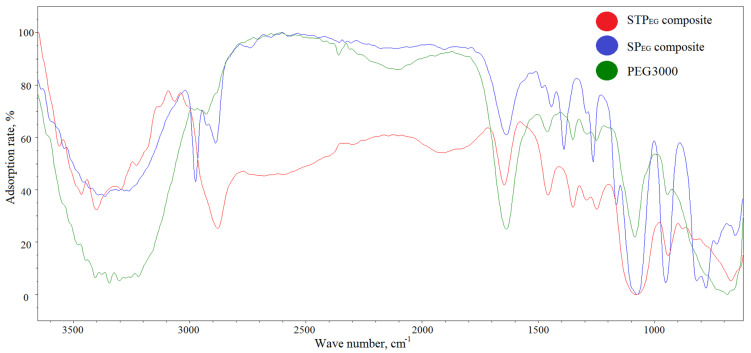
IR spectra of organosilica composites: red line, STP_EG_-composite; blue line, SP_EG_-composite; and green line, PEG3000.

The formation of the aforementioned structures of organosilica materials during sol-gel synthesis is confirmed by IR spectroscopy (Figure 4).

The IR spectra of both samples of organosilica hydrogels display absorption bands specific for polyethylene glycol and silica compounds. For instance, an intense band is observed in the 2870–2890 cm^−1^ area, which is attributed to oscillations of –C-H groups in the main chain of PEG, while 1475–1450 cm^−1^ bands are attributed to oscillations of -CH_2_-groups. However, the intensity of –C-H groups in the 2870–2890 cm^−1^ area is higher in STP_EG_ because the PEG:TEOS mass ratio in these composites is 32 times higher than in SP_EG_-composites. In both spectra of organosilica composites, specific peaks related to symmetric twisting of Si-OH are observed with 1640 cm^−1^ maxima, whereas intense 1070 cm^−1^ absorption peaks are attributed to asymmetric valence oscillations of Si-O-. An intense and broad band at 1100–1050 cm^−1^ characterizes the asymmetric valence oscillations of the Si-O-Si group of the silicate part of the composite; the 1150–1060 cm^−1^ band is specific for C-O-C valence oscillations, and the 970–940 cm^−1^ band is specific for the Si-O-C-group. A broad band in the 3700–3300 cm^–1^ range and a peak at 1625 cm^−1^ correspond to hydroxyl stretching in SiO-H and in the hydroxyl groups of PEG (intra- and intermolecular hydrogen bonds in the polymer). The intensity of the SP_EG_-composite material is higher because the initial content of the polymer in this composite is smaller, leading to a higher degree of interaction with water, as was shown earlier, including in the studies described in the literature.

The specific difference in the IR spectrum of SP_EG_-composite from that of STP_EG_ is the presence of intense absorption bands in the 2970–2980 cm^−1^ and 1380–1390 cm^−1^ regions attributed to the oscillation of the CH_3_-group and 1270–1280 cm^−1^ reflecting Si-C oscillations, which is explained by the presence of MTES precursor derivatives containing non-hydrolysable Si-CH_3_ bonds. Specific peaks like the double maximum at 780–800 cm^−1^ are characteristic for ring-shaped structures composed of [SiO4] tetrahedrons (Figure 3a). The absence of this band in the STP_EG_-composite spectrum demonstrates the impossibility of forming a separate quartz-like silicate fraction at a high polymer fraction (PEG) content, as described above (Figure 3b). The IR spectrum of the STP_EG_-composite is characterized by an absorption band at 1260 cm^−1^, which corresponds to deformation oscillations of the C–OH bond. In the IR spectrum of the SP_EG_-composite this band is absent. The C–OH bond is specific to the terminal groups of PEG. The number of C–OH bonds in the STP_EG_-composite is significantly higher than in the SP_EG_-composite because the organic polymer content is higher (Table 1). Moreover, low-molecular PEG400 was used for the production of STP_EG_-composites, whereas SP_EG_ involved PEG3000, which led to an increased fraction of terminal C–OH groups in STP_EG_ by an extra 7.5 times. The detailed modeling of the composite systems based on the PEG matrix and silica particles revealed that the presence of hydroxyl groups at the ends of PEG chains plays a crucial role in the interaction network formation [79]. A decrease in the hydroxyl group count leads to the formation of a less dense network with lower mechanical strength.

Thus, the organosilica composites derived from different silica precursors and with different ratios of inorganic to organic parts differ in the silicate phase structure and its distribution in the composite, which allows obtaining organosilica nanocomposites with different characteristics.

### 3.2. Morphology and Architecture of Biohybrid Materials on the Base of Immobilized Microorganisms in Organosilica Composites

To immobilize methylotrophic yeast *Ogataea polymorpha* VKM Y-2559 into organosilica composites, all the same stages as for composite synthesis were used, but suspension of the microorganisms was used instead of an aliquot of buffer solution. Biocomposites with embedded yeast were similar to organosilica gels without the microorganisms, but they were non-transparent materials.

**Figure 5:**
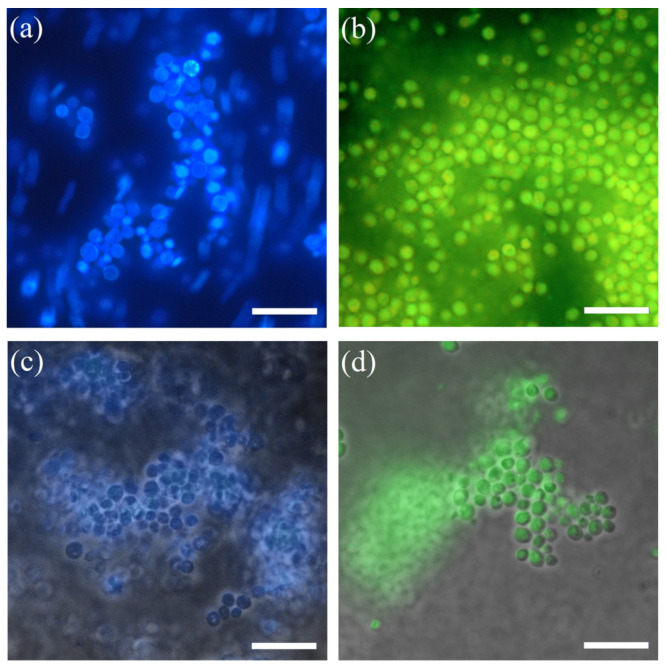
Fluorescence microscopy: (**a**,**b**) suspension of yeast *Ogataea polymorpha* VKM Y-2559; (**c**,**d**) slice of STP_EG_-composite matrix with immobilized yeast *Ogataea polymorpha* VKM Y-2559; (**a**,**c**) cytochemical coloring of the yeast cell wall by fluorescent dye Calcofluor White; and (**b**,**d**) cytochemical coloring of the yeast by fluorescent dye Live/Dead Yeast Viability Kit. The bar represents a 10 μm scale.

The viability of immobilized yeast cells in the STP_EG_-composites was estimated by fluorescent microscopy using a dye system for identification of living and dead cells (Live/Dead Yeast Viability Kit) and a fluorescent reagent specific for yeast surface structures (Calcofluor White M2R) (Figure 5).

Yeast cell surfaces become fluorescent after binding of the specific dye Calcofluor White M2R (Figure 5a), which confirms the integrity of the surface structures of the free microorganisms. Figure 5c,d show the microphotographs of methylotrophic yeast immobilized in a slice of STP_EG_ composite in the presence of fluorescent dyes. Based on the green fluorescence of the methylotrophic yeast (Figure 5d), it can be concluded that all the cells have an intact membrane and are viable. The blue color of the yeast in the slice in the presence of Calcofluor White M2R confirms the integrity of the surface structures of the cell walls of the immobilized microorganisms (Figure 5c). However, some shells around the cells that are absent in the suspension of the microorganisms can be seen on this micrograph. The cells in these shells remain uncolored.

**Figure 6:**
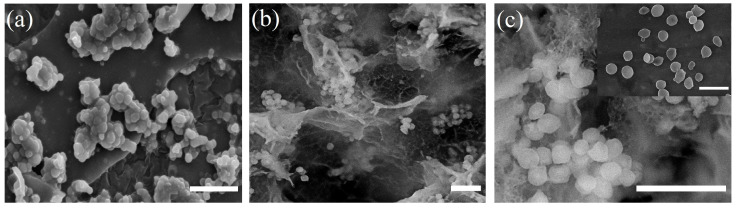
SEM micrograph showing the formation of a 3D structure: (**a**) biohybrid material based on *Ogataea polymorpha* VKM Y-2559 cells encapsulated in an organosilica SP_EG_-composite, (**b**,**c**) biohybrid material based on *Ogataea polymorpha* VKM Y-2559 cells encapsulated in an organosilica STP_EG_-composite, and insert free *Ogataea polymorpha* VKM Y-2559. The bar represents a 5 μm scale.

Structural features of the biohybrid composites were studied by scanning electron microscopy (Figure 6).

Separate cells packed into spherical particles ranging in size from 0.7 to 2 μm can be seen in the biohybrid material based on the methylotrophic yeast immobilized in the SP_EG_-composite (Figure 6a). PEG hydrogels are formed around the cell surface as three-dimensional networks with silica particles. Immobilization of yeast cells into STP_EG_-composite leads to the formation of tighter film-like shells around the cells, which could be explained by the lower water content in the system, as was shown earlier (Figure 6b,c). A similar structure was obtained in our earlier work during the immobilization of yeast in organosilica composites made of TEOS, MTES, and PEG1000 [68]. Such architecture is explained by the application of low-molecular-weight polyethylene glycols, which form linear structures in water solutions.

The structure of organosilica composites is also different: SP_EG_-composites represent monolithic plates, while STP_EG_-composites are a film-like material (Figure 6a,b).

### 3.3. Characterization of the Encapsulated Methylotrophic Yeast as Biocatalysts

The respiratory activity of the encapsulated yeast *Ogataea polymorpha* VKM Y-2559 was studied with an oxygen electrode-based biosensor. A biohybrid composite sample was placed onto the electrode surface as described in Section 2.5. The respiratory activity was recorded after the addition of a substrate (methanol) into the measuring chamber of the biosensor. The rate of oxygen consumption by the immobilized microorganisms depended on the methanol concentration. The rate of oxygen content change after methanol addition (mgO_2_/(dm^3^ × min)) was taken as the sensor’s response. The dependence of the rate of biochemical methanol oxidation by immobilized microorganisms can be mathematically described by the hyperbolic equation of Michaelis–Menten type (1):

(1) $$V=\frac{V_{\max}\times \left[S\right]}{A+\left[S\right]}$$

where V_max_ is the maximal rate of oxygen consumption by immobilized microorganisms, A is a coefficient that is numerically equal to the substrate concentration, at which the rate of the enzymatic reaction reaches half its maximum value, and [S] is the substrate concentration.

**Figure 7:**
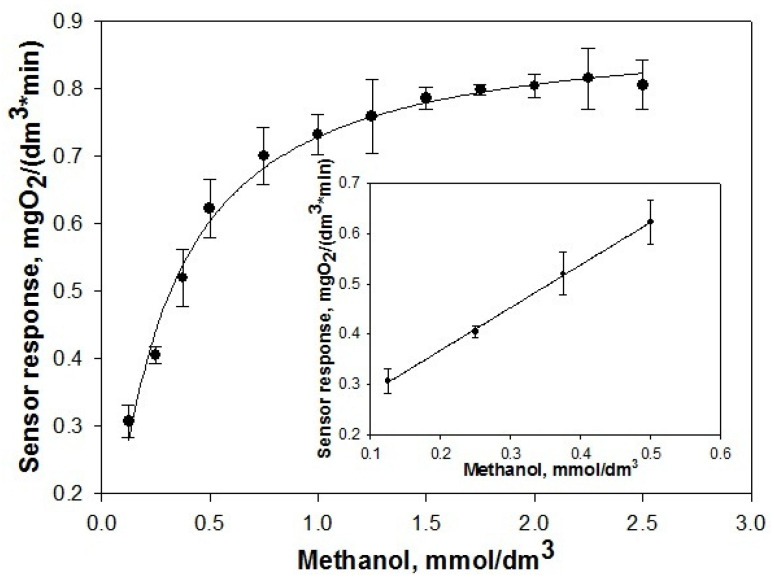
The calibration curve with the standard deviation of the biosensors (n = 7).

For a quantitative estimation of the biocatalyst’s functional effectiveness, the characteristics of a biosensor designed on the basis of the immobilized methylotrophic yeast in the STP_EG_-composite were determined (Figure 7). The obtained results were compared with the characteristics of a biosensor based on the yeast cells encapsulated in SP_EG_-composites.

The quantitative value characterizing the sensitivity of a biosensor is the sensitivity coefficient, which is determined as a derivative of the analytical signal by the concentration of the measured component. The parameters of sensitivity and stability of the biosensors based on the biocatalysts are listed in Table 2.

Comparative analysis of the characteristics of biosensors based on the yeast cells immobilized in STP_EG_- and SP_EG_-composites showed that the biocatalysts are characterized by effective functioning: the biosensor coefficient of sensitivity is 0.85 ± 0.07 mgO_2_ × min^−1^ × mmol^−1^ and 0.87 ± 0.05 mgO_2_ × min^−1^ × mmol^−1^, respectively. As for the other characteristics, a STP_EG_-biocatalyst is a little inferior to a SP_EG_ biocatalyst, which could be caused by the lower water content in the microenvironment of the living cells.

#### 3.3.1. Characterization of the Immobilized Methylotrophic Yeast as Biocatalysts by Biosensor Assessment Technologies after UV Irradiation

Silica materials, particularly glass, are known to be impermeable to shortwave and mediumwave UV radiation. UV radiation is widely used in microbiology, biotechnology, and healthcare for sterilization of equipment, which is why it is so important to understand how effectively the organosilica matrices could protect the living cells under irradiation. To test this factor, immobilized yeast cells were irradiated by UV light in the shortwave region (λ = 254 nm) for 5 h, and after that their respiratory activity was measured with application of biosensor technologies (Table 3).

It turned out that the characteristics of sensitivity and stability of the biocatalyst based on yeast immobilized in the STP_EG_-composite after irradiation decreased by no more than 15% compared to the biocatalyst not exposed to irradiation, which proves the protective properties of the STP_EG_-composite. As it was expected, the silica particles play a crucial role in the UV-protective properties of the composites. Earlier we showed that a SP_EG_-composite provides more effective protection of living cells from UV irradiation, which is caused by the high content of silica in the composite [65].

#### 3.3.2. Effect of Heavy Metal Ions on the Respiratory Activity of the Immobilized Microorganisms

**Figure 8:**
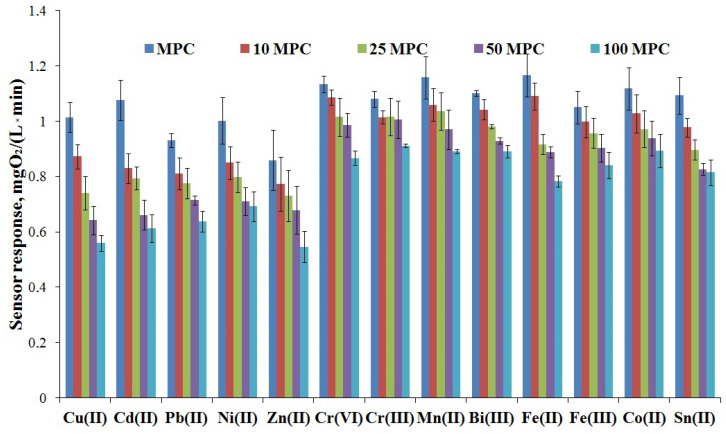
Effect of heavy metal ions on the respiration activity of yeast cells immobilized in organosilica composites (±standard deviation, n = 10).

Heavy metal ions have bactericidal effects. To study the influence of heavy metal ions, a calculated amount of the ions was introduced into the cuvette of the biosensor appliance, corresponding to 1–100 MPC. The measured parameter was the response of the sensor to substrate addition in the presence of heavy metal salts (Figure 8).

As shown in Figure 8, the respiratory activity of microorganisms immobilized in the STP_EG_-composite decreased by 20–30% in the presence of heavy metal ions, whereas the activity of free yeast (in the planktonic state) decreased by 80–90%, as was shown in the study by the authors of [28]. We suggest that the main factor in the protection of the microorganisms from the action of heavy metals is the ability of the composite to retain them by electrostatic interactions within the silica filler and the creation of a partially hydrophobic barrier by the polymer matrix of the composite.

### 3.4. Use of the Biocatalyst as a Biosystem for Methanol-Rich Wastewater Utilization

**Figure 9:**
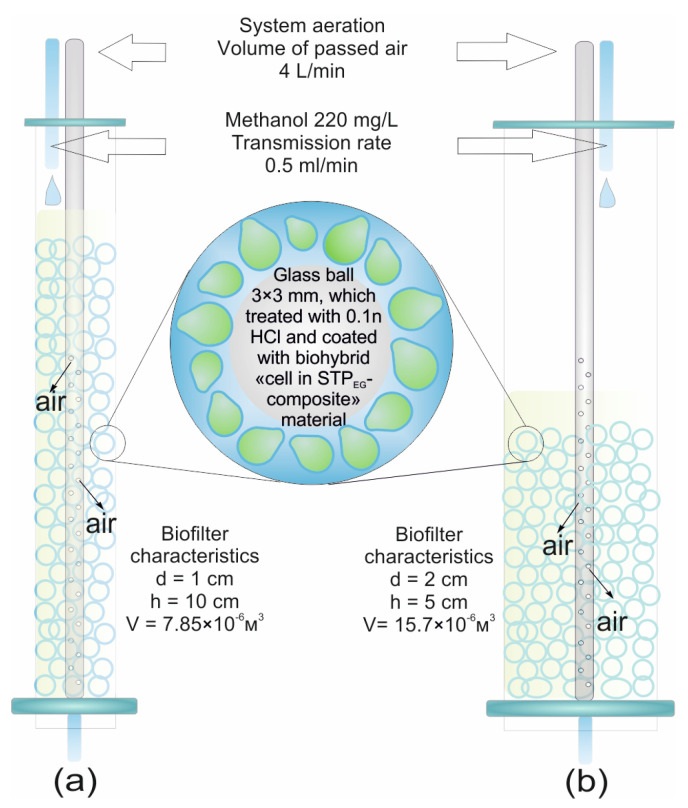
Operational guidelines for trickling biofilter columns: biofilter bed—glass beads of 3.3 mm in diameter with surfaces modified with encapsulated yeast cells; test conditions: biological treatment of wastewater with a methanol concentration of 220 mg/L in (**a**) a long column or (**b**) a short column.

The possibility of using the methylotrophic yeast immobilized in the STP_EG_-composite for methanol utilization was studied in a laboratory model of a column-type trickling biofilter (Figure 9).

The methanol content in the studied model wastewater corresponded to the maximal permitted concentration of methanol in wastewater for biochemical treatment according to Russian normative documents and was equal to 220 mg/L. The ability of the laboratory biofilter to utilize methanol in the model wastewater was studied in two models, with natural and active aeration, at a flow rate of 0.5 mL/min (0.96 L/h × L_filter bed_). Two columns were used, one with a diameter of 10 mm and a height of 100 mm (Figure 9a, hereinafter—long column), and another with a diameter of 20 mm and a bed height of 50 mm (Figure 9b, hereinafter—short column).

**Figure 10:**
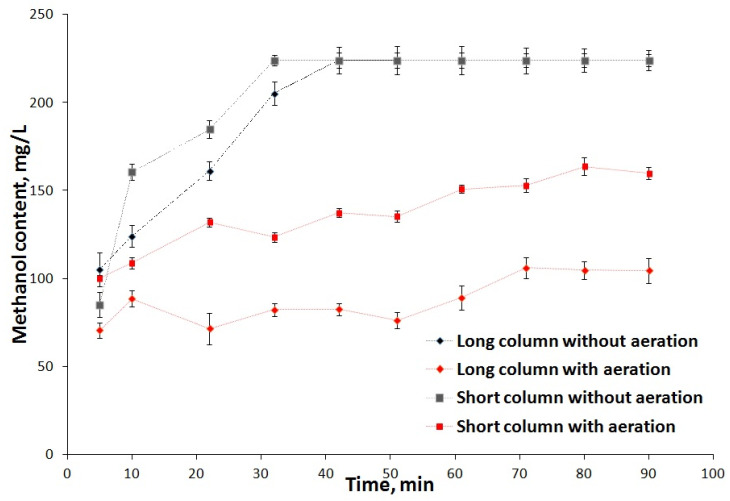
Dependence of methanol content at the outlet of the trickling biofilter column with a biocatalyst based on yeast encapsulated in STP_EG_-composite on the time of operation (standard deviation, n = 10).

Under the conditions of aerobic methanol utilization, the limiting stage is providing enough oxygen to the immobilized cells. A dramatic decrease in the rate of methanol degradation was observed in the first minutes of operation (Figure 10 (without aeration)). After 40–50 min of work, the biofilter completely lost its operational ability. This is related, first of all, to the insufficient concentration of oxygen in the loaded matter, which does not allow the microorganisms to oxidize methanol effectively. The utilization degree under natural (passive) aeration comprised about 10% of the initial methanol quantity in the model wastewater. Under active aeration, the process of methanol oxidation became more effective, and the utilization degree rose by threefold or more (Figure 10 (with aeration)).

Higher efficiency of methanol oxidation was observed in the biofilter based on the longer column because, in this configuration, the height of the loaded bed is greater, so the methanol-containing solution was in contact with the loading material of the biofilter for a longer period of time and the amount of methanol oxidized by the microorganism immobilized increased.

The efficacy of the biofilter operation was determined by its oxidative power (*OP*, gO_2_/m^3^ × series). *OP* is the number of oxygen grams per cycle (90 min) that could be provided by 1 m^3^ of loading material to decrease biological oxygen demand (BOD), and it is calculated by Formula (2):

(2) $$OP=\frac{(BOD_{inc.}-BOD_{pur.})\times Q}{V_{biofilter\;feed}}\;\mathrm{where}$$

*BOD_inc._* is the BOD of incoming wastewater, gO_2_/m^3^; considering that the oxidation of 1 mg of methanol accounts for 0.98 mg of O_2_ and the BOD of the incoming model runoff was 220 mg/L (220 gO_2_/m^3^).

*BOD_pur._* is the BOD of purified wastewater, gO_2_/m^3^; *Q* is the amount of wastewater, in this case 5 × 10^−5^ (m^3^/series); and *V_biofilter feed_* is the biofilter feed volume, 7.85 × 10^−6^ m^3^ for a long column, 15.7 × 10^−6^ m^3^ for a short column.

The characteristics of the biofilter operation are listed in Table 4.

Under passive aeration, the oxidative power per cycle of biofilter operation (90 min) ranged from 75 to 270 gO_2_/(m^3^ × cycle) depending on the biofilter configuration. The active aeration of the biofilters provided a 3-fold increase in their efficacy. The oxidative power was almost 900 gO_2_/(m^3^ × cycle) for the long-column-based biofilter, and the purification degree for model methanol-rich wastewater was 60%, which is a normal value for trickling biofilters. Previously, similar utilization degree values were obtained for biocatalysts based on microorganisms encapsulated in SP_EG_-composite [68].

Thus, methylotrophic yeast immobilized in STP_EG_-composites are effective biocatalysts for the development of wastewater purification biosystems.

## 4. Conclusions

Silicon polyethylene glycol have a significant advantage over typical alkoxysilanes because low-molecular alcohols are not formed during hydrolysis and condensation reactions, which allow for the avoidance of the death of living cells upon immobilization. Silicon polyethylene glycol was first used for the immobilization of living cells. Firstly, we have demonstrated the formation of organosilica shells from STP_EG_-composite over the methylotrophic yeast surface. The microorganisms encapsulated in PEG-silica composites are protected from UV radiation and the toxic action of heavy metal ions, and they can be used as loading bed materials for biofilters in wastewater treatment systems. Biofilters based on the developed biocomposites are characterized by effective utilization of methanol. Under passive aeration, the oxidative power per one cycle of biofilter operation (90 min) was from 75 to 270 gO_2_/(m^3^ × cycle), depending on biofilter configuration. The active aeration of the biofilters provided a 3-fold increase in their efficacy. The oxidative power is almost 900 gO_2_/(m^3^ × cycle) for the long-column-based biofilter and the purification degree for model methanol-rich wastewater is 60%, which is a normal value for trickling biofilters.

Thus, the directed synthesis of organosilica composites and biocomposites of different structures depending on the initial components and their components is possible on the basis of TEOS and PEG in sol-gel synthesis reactions. This should be taken into account when developing biocatalysts based on immobilized into ORMOSIL-composite microorganisms.

## Figures and Tables

**Table 1:** Mass ratio of silica and polymer components in organosilica composites.

Composites	Mass Ratio of Reagents in the Sol-Gel Synthesis
PEG400:TEOS (STP_EG_)	1:0.125 (~90% PEG)
PEG3000:TEOS/MTES (15/85) (SP_EG_)	1:4 (~20% PEG)

**Table 2:** The characteristics of biosensors based on the microorganisms immobilized in organosilica composites.

Parameter	STP_EG_	SP_EG_ [66,68]
Sensitivity coefficient, mgO_2_ × min^−1^ × mmol^−1^	0.85 ± 0.08 *	0.87 ± 0.05 *
Relative standard deviation, %	10	3
Long-term stability, days	10	15

* Confidence interval, n = 5, and *p* = 0.95.

**Table 3:** The characteristics of the biosensor based on immobilized yeast cells in an organosilica STP_EG_-composite after UV irradiation.

Parameter	Before UV Irradiation	After UV Irradiation
Sensitivity coefficient, mgO_2_ × min^−1^ × mmol^−1^	0.85 ± 0.07	0.73 ± 0.08
Relative standard deviation, %	10	8
Long-term stability, days	10	9

**Table 4:** Characteristics of the biofilter with the loading bed material made of methylotrophic yeast encapsulated in STP_EG_-composite under natural (passive) and active aeration.

Column Configuration	Purification Degree, %	Oxidative Power, gO_2_/(m^3^ × Cycle)
Passive aeration		
Long column (d = 1 cm; h = 10 cm)	13 ± 1 *	268 ± 1 *
Short column (d = 2 cm; h = 5 cm)	10 ± 1	75 ± 1
Active aeration		
Long column (d = 1 cm; h = 10 cm)	58 ± 1	898 ± 1
Short column (d = 2 cm; h = 5 cm)	31 ± 1	248 ± 1

* Confidence interval, n = 5, and *p* = 0.95.

## Data Availability

Not applicable.
